# Identification of respiratory chain complex I deficiency due to *NDUFA5* variants as a novel cause of infantile fatal disease

**DOI:** 10.1016/j.gendis.2025.101920

**Published:** 2025-11-06

**Authors:** Yang Wang, Xi Yang, Xue Yan, Yumin Zhu, Xue Xia, Ting Li, Yi Liao, Bingkun Lei, Jingmin Yang, Deyuan Li

**Affiliations:** a Department of Pediatric, West China Second University Hospital, Sichuan University, Chengdu, Sichuan 610041, China; b Key Laboratory of Birth Defects and Related Diseases of Women and Children, Sichuan University, Ministry of Education, Chengdu, Sichuan 610041, China; c Shanghai WeHealth Biomedical Technology Co., Ltd., Shanghai 201315, China; d Department of Radiology, West China Second University Hospital, Sichuan University, Chengdu, Sichuan 610041, China; e Obstetrics and Gynecology Hospital, Fudan University, Shanghai Key Lab of Reproduction and Development, Shanghai Key Lab of Female Reproductive Endocrine Related Diseases, Shanghai 200433, China; f Key Laboratory of Birth Defects and Reproductive Health of National Health and Family Planning Commission (Chongqing Key Laboratory of Birth Defects and Reproductive Health, Chongqing Population and Family Planning, Science and Technology Research Institute), Chongqing 400020, China; g State Key Laboratory of Genetic Engineering, School of Life Sciences, Fudan University, Shanghai 2004382, China

Mitochondrial complex I (CI), also known as NADH-ubiquinone oxidoreductase, represents the largest and most intricate component of the mitochondrial oxidative phosphorylation (OXPHOS) system, which is composed of 44 different subunits in humans and is assembled from the 14 core subunits.^1^ CI, a key component of the electron transport chain, represents a crucial site for the physiological production of reactive oxygen species (ROS) during cellular respiration, commonly recognized as being intimately linked to the oxidative damage that occurs subsequent to hypoxic exposure.^2^ Complex I is a distinctive boot-shaped structure composed of 14 core protein subunits organized into two main domains. The N module, located at the top of the matrix arm, assumes a pivotal role in catalyzing the oxidation of NADH. Meanwhile, the Q module functions as a vital connector between the substrate arm and the membrane arm, facilitating the transfer of electrons. The Q module comprises a set of proteins, including NDUFS2, NDUFS3, NDUFS7, NDUFS8, NDUFA9, and notably NDUFA5, which serves as a nuclear-encoded structural accessory subunit positioned within the peripheral segment of Complex I’s Q model. Previous studies have conclusively demonstrated that the lack of the *NDUFA5* subunit leads to the loss of the membrane arm subcomplex. One of the interacting subunits of NDUFA5 is NDUFS2, which is a core subunit of mitochondrial complex I and is encoded by nuclear DNA. NDUFS2 plays a crucial role in the catalytic activity and assembly of mitochondrial complex I.^3^^,^^4^ Mitochondrial diseases are a group of inherited metabolic disorders with a high degree of clinical and genetic heterogeneity.^5^ Epidemiological data revealed that CI deficiency carries a grave prognosis, with approximately 50% of affected patients succumbing within the initial two years of life, while merely a quarter manage to reach 10 years old. Lactic acidosis is a common feature of the disease caused by CI variants and is accompanied by other symptoms, such as cardiomyopathy or leukodystrophy. In this study, we described a four-month-old child with progressive neurological deficits, postpartum lactic acidosis, encephalopathy, developmental abnormalities, metabolic abnormalities, and respiratory failure. Whole genome sequencing (WGS) identified two heterozygous variants of the *NDUFA5* gene. A series of *in vitro* functional experiments were conducted and demonstrated that the *NDUFA5* gene was associated with CI functional defects for the first time.

A 4-month-old male infant was admitted to the neonatal intensive care unit (NICU) of West China Second University Hospital, Sichuan University, due to respiratory infection, presenting with dyspnea, altered mental status, seizures, and shock. Besides, the proband had delayed development after birth. His parents and elder brother were healthy, and the parents denied any history of consanguinity. There was no recorded history of hereditary diseases within the family.

Further examination revealed severe metabolic disorders, high lactate 6.6 mmol/L (0.7–3.0 mmol/L), pyruvate 214 μmol/L (20–100 μmol/L) and high blood ammonia 71 μmol/L (10–47 μmol/L). Blood metabolic screening results showed elevated levels of Ala 590 μM (18–266 μM), Gly 594.4 μM (26–206.4 μM), Ser 179 μM (16–132 μM), Phe/Tyr 3.0 (0.3–2.3), C18:1 0.59 μM (0.00–0.34 μM), C4–OH 1.49 μM (0.00–0.29 μM), and C5DC/C8 4.08 (0–1.10). Blood routine, liver and kidney function, myocardial injury markers, muscle enzymes, cerebrospinal fluid examination, blood glucose, and echocardiography showed no significant abnormalities.

Brain magnetic resonance imaging (MRI) showed abnormal symmetrical signal of white matter in both hemispheres of the brain, delayed myelination of white matter; bilateral frontal and temporal extra brain spaces widened, bilateral lateral fissure pools widened, and bilateral ventricles slightly blunted (Fig. S1). Based on the above examination, it is clinically suspected to be mitochondrial encephalopathy.

The patient was managed with invasive mechanical ventilation, shock reversal, hemoperfusion to correct the deranged metabolic state, supplementation with various metabolic substrates, antibiotics, and neuroprotection. Following these interventions, the infant’s respiratory function and consciousness improved significantly. The results of blood gas analysis and blood metabolic results were normalized. A follow-up brain CT revealed an expansion of the lateral ventricles compared to the initial scan, with slightly decreased density in the bilateral frontal lobe white matter. Unfortunately, the infant experienced diarrhea and vomiting during hospitalization, followed by coughing with noticeable pharyngeal rales. The infant then exhibited progressive signs of respiratory distress and irritability. The blood gas analysis indicated a high anion gap metabolic acidosis with an elevated lactate level of 8.30 mmol/L. Tragically, the infant ultimately succumbed to the inability to correct the deranged intracellular environment, along with respiratory and circulatory failure, leading to his unfortunate demise at the age of 6 months.

To explore the potential genetic etiology of the patient, we performed WGS on the proband. The relevance shown in Table S1, two heterozygous variants were detected in *NDUFA5* (NM_005000.5): M1: c.335G > A:p.W112∗ and M2: c.67-2A > G. As the relevance of the gene to the disease was not clear and the two variants mentioned above have not been previously reported, they were absent from the gnomAD_EAS population database (PM2_Supporting) and were predicted to be damaging by multiple *in silico* tools (PP3). Consequently, both variants were classified as variants of uncertain significance (VUS) according to the American College of Medical Genetics (ACMG) guidelines (Table S2). Sanger sequencing revealed that the variant c.335G > A in *NDUFA5* was paternally inherited, and the variant c.67-2A > G was maternal origin (Fig. 1A and B).

**Figure 1:**
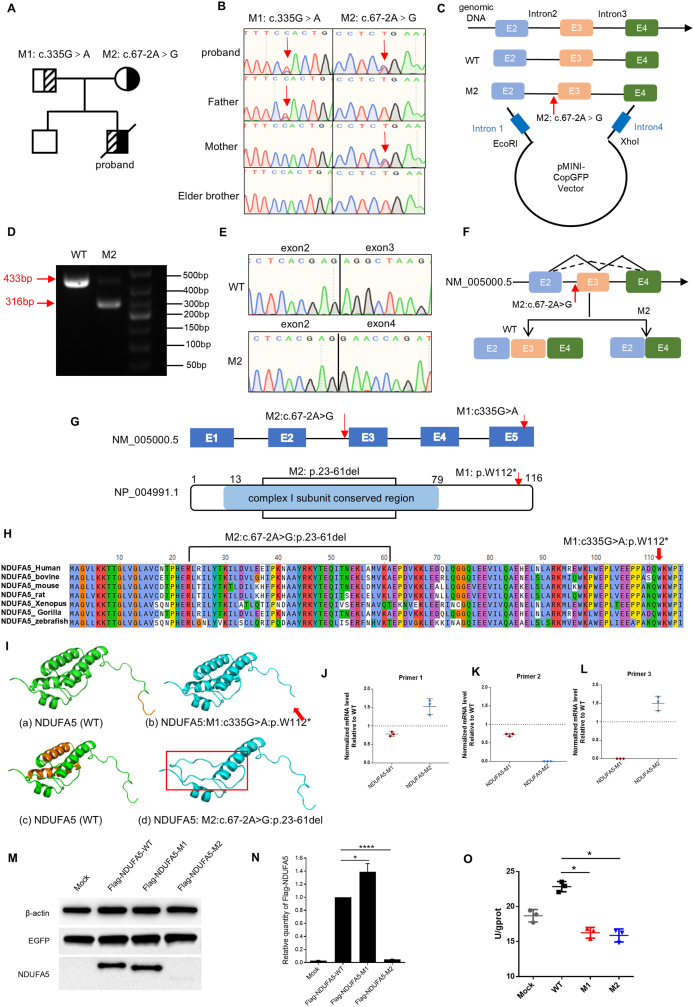
Identification of two variants in *NDUFA5* and the results of *in vitro* functional experiments. **(A)** Pedigree of the proband. M1 represents c.335G > A, and M2 represents c.67-2A > G. The carrier filled in half black or half shadow. The proband was compound heterozygous. **(B)** Sanger sequencing of the proband and his family. The red arrows represent the locus of the variants. **(C)** DNA fragments, including those from the mutant site and WT site, were cleaved from the gDNA of healthy individuals and probands via PCR. The target sequence of exons and its flanking introns were inserted into the minigene vector. **(D)** Analysis of minigene transcript RT-PCR products via electrophoresis. The WT exhibited a band of 433 bp, and the M2 exhibited a band of 433 bp and a band of 316 bp. **(E)** Sanger sequencing of PCR products. **(F)** Diagrammatic representation of the genomic locus containing the c.67-2A > G variant in the *NDUFA5* gene, illustrating the resulting aberrant mRNA splicing pattern. **(G)** Gene structure of *NDUFA5* and distribution of identified variants. The protein structure highlights the complex I subunit conserved region domain in light blue. **(H)** Interspecies alignment of the NDUFA5 and amino acid conservation around the NDUFA5 protein. **(I)** Structural homology modeling of WT (green) and mutant NDUFA5 (blue) proteins. (a, c) WT protein (amino acids 1–116). Orange highlights the amino acid sequences that differ between the WT and mutant types of NDUFA5. (b, d) Mutant NDUFA5 protein. The red box and red arrow indicate abnormal areas. **(J**–**L)** The expression level of *NDUFA5* mRNA was analyzed by quantitative real-time (RT)-PCR using reverse-transcribed cDNA from 293T cells transfected with WT/M1/M2 plasmids. **(M, N)** NDUFA5 protein expression levels of M1 and M2. β-actin was used as a loading control. EGFP was used as a transfection control. Densitometry data are expressed as the mean ± SD, *n* = 3. ∗*P* < 0.05, and ∗∗∗∗*P* < 0.0001. **(O)** Mitochondrial complex I activity assay of 293T cells transfected with WT and M2 plasmids. Data are expressed as the mean ± SD, *n* = 3, ∗*P* < 0.05.

To elucidate the functional significance of the splicing variant c.67-2A > G, we designed and implemented a minigene assay for both the wild-type and mutant sequences of the *NDUFA5* exon 2 to exon 4. The constructed plasmids pMini-CopGFP-wt and pMini-CopGFP-M2 were then transiently transfected into 293T cells, respectively (Fig. 1C).

Agarose gel electrophoresis results showed that cells transfected with the wild type (WT) vector yielded a single 433 bp amplicon corresponding to *NDUFA5* exons 2–4, while cells transfected with the mutant vector displayed two distinct bands, a 433 bp fragment representing the normal amplicon and a 316 bp mutant amplicon (Fig. 1D). Sanger sequencing revealed the skipping of exon 3 in the mutant type (Fig. 1E). Hence, these findings conclusively illustrated that the c.67-2A > G variant disrupts exon 3 of *NDUFA5*, which results in a loss of 117 bp and a loss of 39 amino acids, p.23-61del (Fig. 1F).

As the *NDUFA5* gene consists of five exons, we evaluated the impact of the *NDUFA5* variants on the encoded protein through *in silico* methods. Bioinformatics analysis revealed that the affected amino acid residues are highly conserved across diverse species, suggesting their critical role in maintaining NDUFA5 protein function (Fig. 1G and H). The splicing variant of c.67-2A > G resulted in a deletion of arginine from position 23 to alanine at position 61 of the NDUFA5 protein, which was predicted to be present in a conserved region of mitochondrial complex I. The predicted structures of the NDUFA5 WT and mutant proteins showed substantial differences (Fig. 1I).

Next, we constructed overexpression plasmids pcDNA3.1-3 × flag-*NDUFA5*-WT/M1/M2 and transfected them into 293T cells, respectively, to further confirm whether they simultaneously affect protein function (Fig. S2). After 24 h of transfection, RT-PCR was used to analyze the transcription levels of mutant and control *NDUFA5*. We designed three pairs of primers based on the different variant positions of M1 and M2. The primer sequences and positions are shown in Table S3 and Figure S3. Overall, compared to the WT group, M1 down-regulated the mRNA expression of *NDUFA5*, while M2 led to up-regulation of mRNA expression (Fig. 1J–L). NDUFA5 protein was drastically decreased in M2 to a level close to the detection limit, while the protein expression level in M1 slightly increased (Fig. 1M and N). The decreased protein level and increased mRNA expression level of M2 may be a result of negative feedback regulation.

To understand the molecular implications of the two variants in more detail, we analyzed the bioactivity of CI in M1-and M2-overexpressing 293T cells. Compared with the WT, both variants showed reduced CI activity (Fig. 1O).

The results of *in vitro* functional assays suggested that the two variants affect *NDUFA5* mRNA expression, protein structure and expression levels, and decreased the activity of CI. The results further indicated that both variants represent loss-of-function variants. Accordingly, PS3 evidence can be applied, supporting the reclassification of VUS as potentially pathogenic.

In conclusion, we reported compound heterozygous variants of *NDUFA5* in a patient for the first time and demonstrated the pathogenicity of the two variants by *in vitro* experiments. Furthermore, we demonstrated that compound heterozygous pathogenic variants in the *NDUFA5* gene are important for the development and function of CI, providing additional evidence for the contribution of respiratory chain CI deficiency and facilitating its diagnosis and treatment.

## CRediT authorship contribution statement

**Yang Wang:** Writing – original draft, Data curation. **Xi Yang:** Writing – original draft, Methodology, Conceptualization. **Xue Yan:** Writing – original draft, Methodology. **Yumin Zhu:** Visualization, Investigation. **Xue Xia:** Visualization, Investigation. **Ting Li:** Visualization. **Yi Liao:** Data curation. **Bingkun Lei:** Writing – review & editing. **Jingmin Yang:** Writing – review & editing, Methodology, Funding acquisition, Conceptualization. **Deyuan Li:** Writing – review & editing, Supervision, Funding acquisition, Conceptualization.

## Ethics declaration

This study was approved by the Ethics Committee of West China Second University Hospital of Sichuan University (No. 2020-111) and the informed consent was signed by the parents of the proband.

## Data availability

The datasets of the study are available from the corresponding author on reasonable request.

## Funding

This work was supported by Sichuan Science and Technology Program (China) (No. 2023ZYD0124), 10.13039/501100018542Natural Science Foundation of Sichuan Province, China (No. 24NSFSC2401) and Chongqing Research Institute Performance Incentive and Guidance Special Project (China) (No. cstc2022jxj10127).

## Conflict of interests

The authors declare that they have no competing interests.
