# Supplementary material for: Identification of respiratory chain complex I deficiency due to NDUFA5 variants as a novel cause of infantile fatal disease
Source: Genes Dis. 2025 Nov 6;13(4):101920. doi: 10.1016/j.gendis.2025.101920 (PMC12995688; doi:10.1016/j.gendis.2025.101920)
Supplement: Multimedia component 1 [file mmc1.docx]

**Identification of Respiratory chain complex I deficiency due to *NDUFA5* variants as a novel cause of infantile fatal disease**

**Supplementary Information**

The supplementary information consists of methods and materials, 3 supplementary figures, and 5 supplementary tables.

**Materials and methods**

**Ethical compliance and sample collection**

This study was approved by the Ethics Committee of West China Second University Hospital of Sichuan University (No.2020-111) and the informed consent was signed by the parents of the proband. Blood samples were collected from the proband and the family members after obtaining written informed consent for diagnostic testing.

**Whole genome sequencing and data analysis**

Genomic DNA extraction was performed using the QIAamp DNA Blood Mini Kit (Qiagen, Germany) from peripheral blood samples collected from affected individuals and their first-degree relatives. DNA libraries were prepared with the TruSeq Nano DNA HT Sample prep Kit (Illumina, USA) and sequenced on HiSeq X platform to produce 150bp paired-end reads, achieving a minimum coverage depth of 30×. The resulting sequencing data were aligned against the GRCh38/hg38 human reference genome using SpeedSeq alignment tools.^1^ Variants calling, including single nucleotide variants (SNVs) and insertion-deletion polymorphisms (InDels), was performed through the HaplotypeCaller algorithm implemented in the Genome Analysis Toolkit, following established best practices for variant analysis.^2^ Structural variants (SVs) were identified through SpeedSeq analysis, followed by comprehensive functional annotation of all detected variants utilizing the ANNOVAR platform for genomic interpretation.^3^

To be classified as pathogenic, SNVs/InDels must satisfy the following criteria:  1) exhibit an allele frequency below 0.01 in both the GnomAD and GnomAD_EAS databases; 2) be situated in exonic or splicing regions and predicted to cause a premature stop codon, frameshift insertion, or deletion, or be located at canonical splice sites; 3) or predicted to be pathogenic at least one of the 6 following programs: Mutation Taster, Polyphen-2, SIFT, CADD, DANN and spliceAI. SVs affecting coding region were classified as pathogenic if their allele frequency were below 0.01 in the internal database.

**Sanger sequencing**

Polymerase chain reaction (PCR) protocol: a pre-denaturation at 95°C for 5 minutes, followed by 35 cycles of denaturation (95°C, 30s), annealing (60°C, 30s), and elongation (72°C, 30s), with a final extension step at 72°C for 5 minutes. Sanger sequencing of the *NDUFA5* amplicon was performed on the ABI3730XL platform (Applied Biosystems, USA), and the sequences were compared to reference genome using Codon Code Aligner.

**3D protein structure modeling**

To predict the protein structure of NDUFA5, the AlphaFold database (https://alphafold.com/) was employed, following the methodology outlined in previous research.^4^ The 3D structure of the human NDUFA5 protein (UniProt ID: Q16718) was obtained from the AlphaFold Database and employed as a template for structural modeling. Both the wild-type NDUFA5 and mutant-type were predicted using SWISS-MODEL (<https://swissmodel.expasy.org/interactive>).  The resulting tertiary structures were subsequently visualized, analyzed, and structurally aligned using the PyMOL molecular graphics system.

**Minigene assay**

Briefly, intronic regions containing the variant or control DNA was amplified and cloned into the minigene. The primers for fragment cloning amplification were listed in Table S4. The amplified products were then inserted into pMini-CopGFP vector (Gengsi bio.tech, China) with EcoRI/XhoI restriction sites using the Hieff Clone One Step Cloning Kit (Yeasen, China). Following verification, both wild-type and mutant minigene plasmids were transfected into 293T cells for 48 hours. Total RNA was isolated using TRIzol reagent (TAKARA, Japan) to conduct RT-PCR and Sanger sequencing. Primers for RT-PCR were listed in Table S5. The resulting PCR products were analyzed by agarose gel electrophoresis to determine fragment lengths, while splicing patterns were confirmed by Sanger sequencing.

**Expression vector construction and Cell transfection**

Full length of wild-type (WT) human *NDUFA5* gene, c.335G＞A mutant, and c.67-2A>G mutant were chemically synthesized (Transheepbio, China). Then the fragments were subsequently cloned into the eukaryotic expression vector pcDNA3.1 respectively, which were named pcDNA3.1-NDUFA5, pCDNA3.1-NDUFA5-M1 and pCDNA3.1-NDUFA5-M2 (Figure S2). The recombinant plasmids were confirmed by DNA sequencing technology. The primers were: forward-5’-CGCAAATGGGCGGTAGGCGTG-3’, and reverse-5’-CAGGGTCAAGGAAGGCAC-3’.

293T cells were cultured in Dulbecco's modified Eagle's medium (DMEM) supplemented with 10% fetal bovine serum (FBS) and maintained at 37℃ in a humidified atmosphere containing 5% CO_2_. Before transfection, cells were cultured in 6-well plates until reaching 50%-60% confluency and then transiently transfected with the *NDUFA5* expression constructs. An empty pcDNA3.1 vector was transfected as mock control. Moreover, each group was co-transfected with a TE159-EGFP vector, with a ratio of 9:1. Cells were harvested 40h after transfection, and divided into three parts for western blotting, mRNA expression analysis, and mitochondrial complex I activity assay, respectively. All transfection experiments were performed in triplicate using the Lipofectamine™ 3000 reagent (Invitrogen, USA) according to the manufacturer’s instructions.

**RNA isolation and mRNA expression analysis**

Total RNA isolation from 293T cells was performed following the TRIzol reagent protocol (Thermo Scientific, USA) and 500 ng of RNA was further used for reverse transcription using the 1st Strand cDNA Synthesis Kit (YEASEN, China). Quantitative PCR analysis was performed using the SYBR qPCR SuperMix Plus (Novoprotein, China). The specificities of the PCR products were investigated via melting curve analysis. β-Actin served as the internal control and the relative mRNA levels were quantified using the 2^−ΔΔCt^ method. We designed three pairs of primers, and the positions and sequences of the primers are shown in Table S3 and Figure S3.

**Western blotting**

Cells were lysed on ice in RIPA buffer (Thermo Scientific, USA) containing the Protease Inhibitor Cocktail (Merck, Germany) for 30 min and centrifuged (12,000 × g) for 10 min. Protein samples were denatured in SDS-PAGE sample loading buffer at 95℃ for 10 minutes. Subsequently, the denatured proteins were electrophoretically separated on 4%-20% SDS-PAGE gels (GenScript, China), and then transferred to PVDF membranes (Bio-Rad, USA).  Following transfer, the membranes were blocked with 5% fat-free milk in Tris-buffered saline with Tween-20 (TBST) for 1 hour at room temperature. The blocked membranes were incubated with primary antibodies specific to target proteins at 4°C overnight, followed by incubation with secondary antibodies at room temperature for 1 hour. Immunoreactive signal was visualized by ECL luminescent liquid (Share-bio, China) and then subjected to densitometry measurement with Image J. The related antibodies we used included HRP-labeled anti-β-actin (Share-bio, China, Cat. No. SB-AB2001), HRP-labeled anti-FLAG-tag (Beyotime, China, Cat. AF2855), anti-GFP (Beyotime, China, Cat. No. AG281), and HRP-labeled goat anti-mouse IgG (Beyotime, China, Cat. No. A0216).

**Mitochondrial complex I activity assay**

Mitochondrial complex I activity was quantified using the Cell Mitochondrial Complex I (NADH-CoQ Reductase) Activity Assay Kit (Elabscience, China) following the manufacturer's protocol. Collect 1 × 10^6 cell, add 200 μL of extraction solution and 4 μL of protease inhibitor, and shake to mix. The mixture was broken by sonication (ice bath was used for this step), sonicated for 5 s with 10 s intervals and repeated 15 times). After that, 10,000 ×g low-temperature centrifugated for 3 min, the precipitate was discarded and the supernatant was taken to be measured, and a portion of the supernatant was retained to be used for protein concentration determination. The reaction mixture was immediately transferred to a prewarmed (37℃) spectrophotometer (SuPerMax 3100, Flash, China). The absorbance of reaction mixture was measured at 340 nm for mitochondrial complex I. Protein concentrations were measured using BCA Protein Assay Kit (Elabscience, China. The specific activity of mitochondrial complex I was calculated and expressed as μmol/min/g protein (U/gprot).

**Statistics analysis**

All quantitative data were performed in triplicates at least and presented as mean ± SEM. Statistical analyses were performed using unpaired two-tailed Student's *t*-tests to compare differences between experimental groups. *P* < 0.05 is considered a significant difference. * *P* < 0.05, ** *P* < 0.01, *** *P* < 0.001, and **** represents *P* < 0.0001.

**Supplementary Figures**


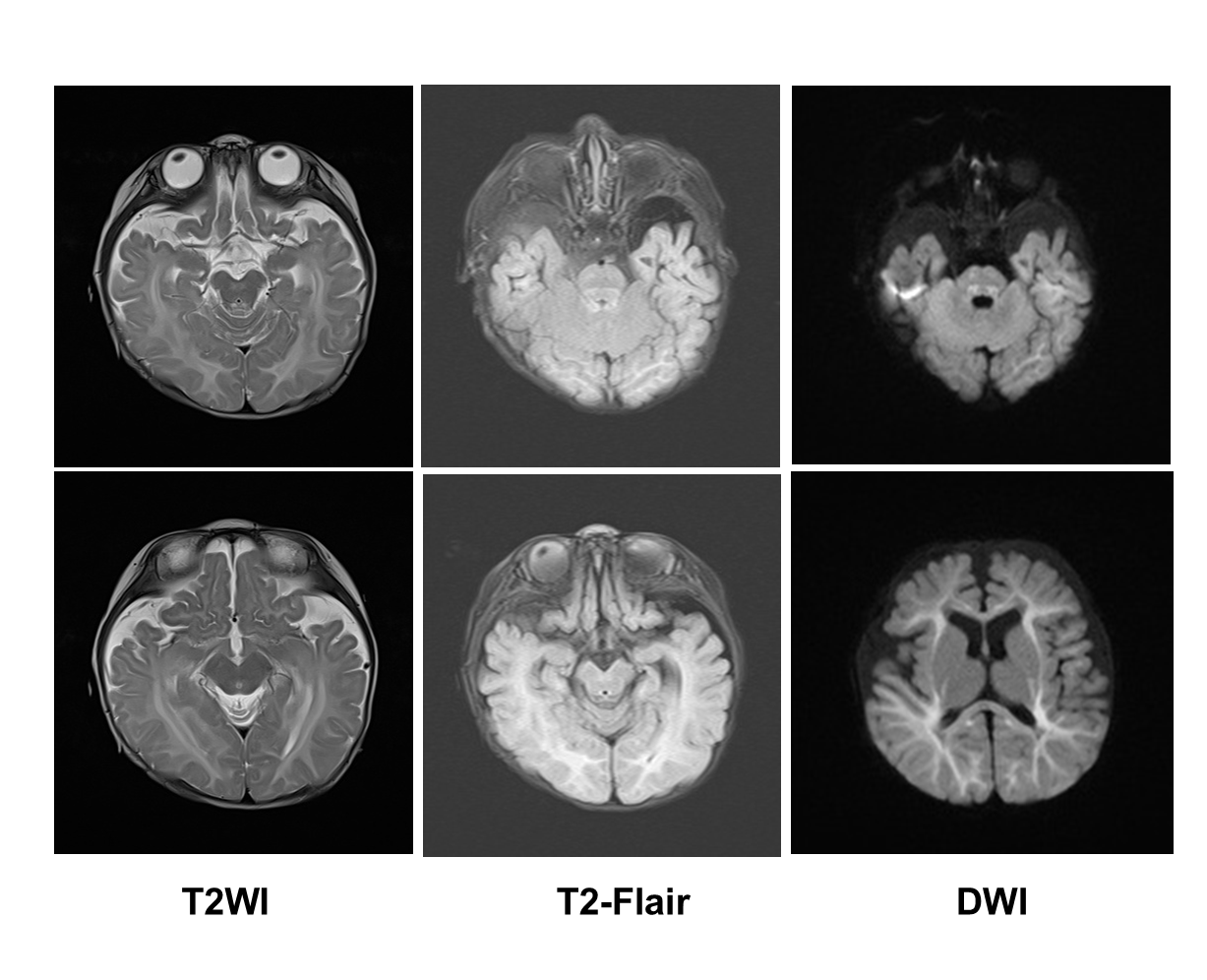


Figure S1. The MRI of the proband.

Magnetic resonance imaging (MRI) of the proband at the age of 4 months. Abnormal symmetrical signal of white matter in both hemispheres of the brain.


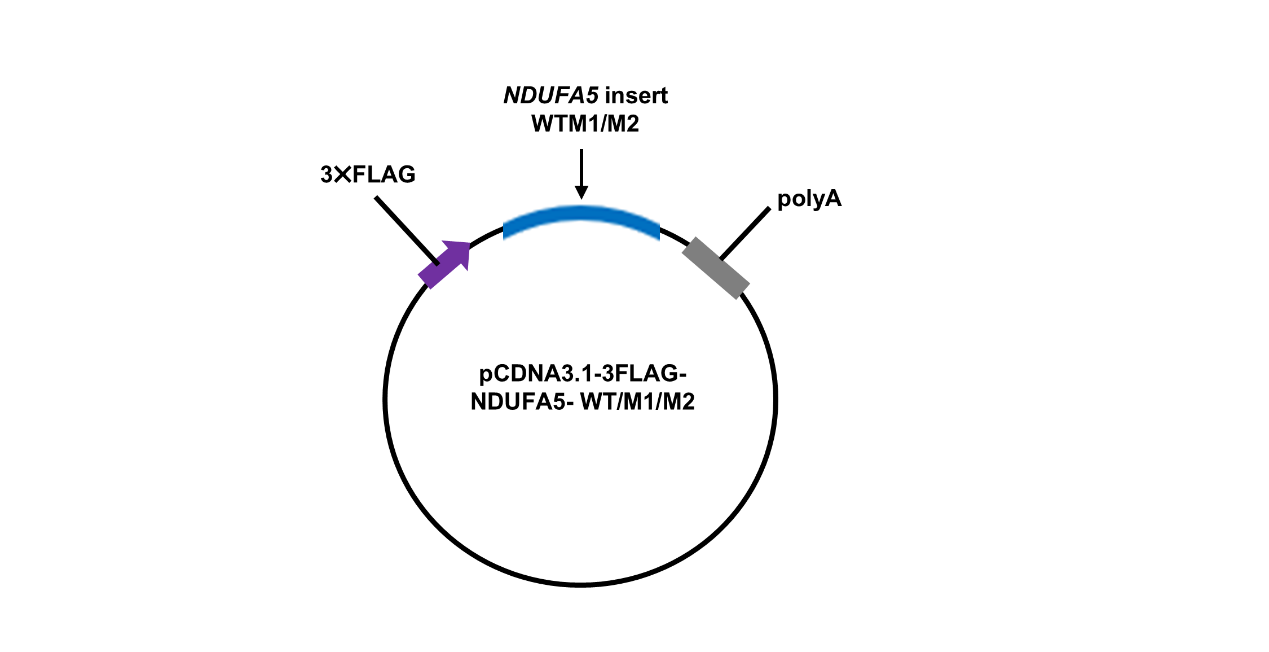


Figure S2. The structure of overexpression plasmids pcDNA3.1-3×flag-*NDUFA5*-WT/M1/M2


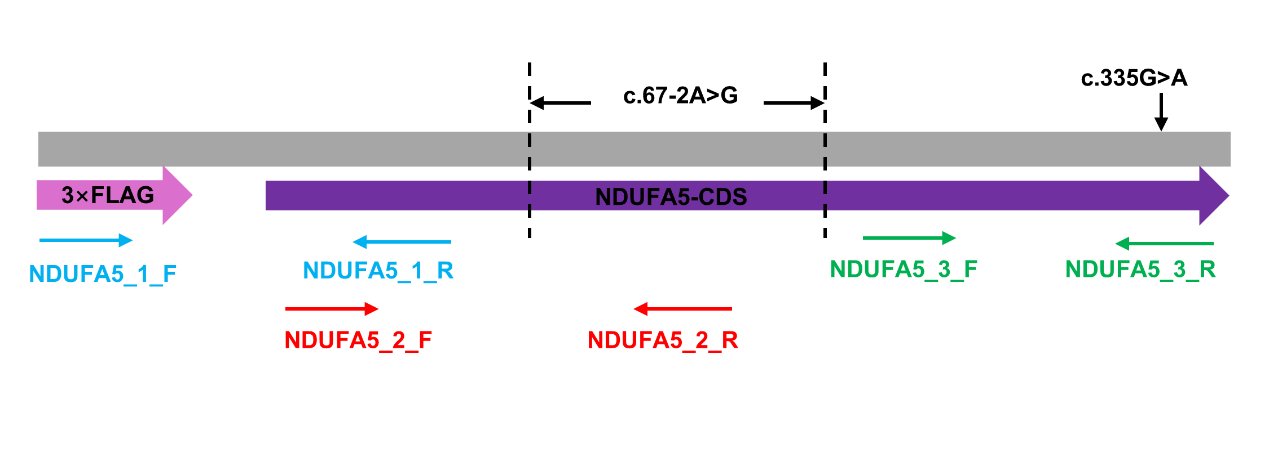


Figure S3. The locations of three pairs of primers

**Supplementary Tables**

Table S1. Overview of the identified *NDUFA5* variants

| variants | cDNA  change^a^ | Protein  change | Mutation type | genotype | ACMG  category | Minor allele frequency^b^ | | Conservation^c^ | | Functional prediction^d^ | | | |
| --- | --- | --- | --- | --- | --- | --- | --- | --- | --- | --- | --- | --- | --- |
|  |  |  |  |  |  | gnomAD_ALL | gnomAD_EAS | PhastCons | Phylop | Mutation  taster | CADD | DANN | SpliceAI |
| 1 | c.335G>A | p.Trp112Ter | nonsense | heterozygous | VUS | Not found | Not found | 1 | 6.142 | Disease causing | 7.769 | 0.993 | - |
| 2 | c.67-2A>G | - | Splicing | heterozygous | VUS | Not found | Not found | 1 | 5.969 | Disease causing | 5.73 | 0.995 | 0.98 |

a The GenBank accession number of *NDUFA5* is NM_005000.5

b Minor allele frequencies were estimated according to the database of the gnomAD_ALL and gnomAD_EAS.

c The more conserved the position, the closer is the Phastcons score to 1, Positive score represents predicted conserved site by Phylop, while larger value means higher conservation.

d Mutation assessment by the MutationTaster, CADD, DANN and SpliceAI tools. Higher CADD and DANN scores suggest that variants are more likely to have deleterious effects. CADD cutoff is usually set as 4, while 0.93 is for the DANN cutoff

Table S2. ACMG classification and evidence for the two variants

| variant | evidence | description | ACMG classification |
| --- | --- | --- | --- |
| c.335G>A | PM2_Supporting | Absent from gnomAD_all and gnomAD_EAS database | VUS |
|  | PP3 | Multiple bioinformatics software predict that this variant may have harmful effects on gene or gene products, |  |
| c.67-2A>G | PM2_Supporting | Absent from gnomAD_all and gnomAD_EAS database | VUS |
|  | PP3 | Multiple bioinformatics software predict that this variant may have harmful effects on gene or gene products |  |

Table S3. Primer sequences for RT- PCR analysis of mRNA expression of *NDUFA5*

|  | Primer-F (5’-3’) | Primer-R (5’-3’) |
| --- | --- | --- |
| NDUFA5_1 | GACTACAAAGACCATGACGGTGATTATAAAGAT | CCAGTGGTCTTCTTCAGCACAC |
| NDUFA5_2 | TATGGCGGGTGTGCTGAAGAAG | TGTTCTGTATACTTTCTATATGCTGCATTTTTAGGGA |
| NDUFA5_3 | GAACCAGATGTTAAAAAATTAGAAGACCAACTTCAAG | AGTTATATTGGCCATTTCC |
| β-Actin | GTGGACATCCGCAAAGAC | AAAGGGTGTAACGCAACTAA |

Table S4. The primers for fragment cloning amplification

|  | Primer-F (5’-3’) | Primer-R (5’-3’) |
| --- | --- | --- |
| WT-1 | CATGGACGAGCTGTACAAGCTCGAGA  CCACTGGCCTTGTGGGATT | CCAGACTGGGAGTCCTGAAGCCCAGTAA |
| WT-2 | TTCAGGACTCCCAGTCTGGCCAACATGGCA | TGTTCTGGAGTGCCTGTGGTCTCAGCTACTT |
| WT-3 | CCACAGGCACTCCAGAACAACAAAACCCTTGC | CGCGGTACCGTCGACTGCAGAATTCTTACTGAAGAATCACCTCTTCTA |
| MUT | GTATGGTTTGTCTTTTCGGAGGCTAAGAATATTGTACAC | CCTCCGAAAAGACAAACCATACAAATTTCCATAGG |

Table S5. Primer sequences for RT-PCR analysis of minigene assay

| Primer-F (5’-3’) | Primer-R (5’-3’) |
| --- | --- |
| CCCGACAACCACTACCTGAG | ACCTCTACAAATGTGGTATGGC |

**Reference**

1. Chiang C, Layer RM, Faust GG, et al. SpeedSeq: ultra-fast personal genome analysis and interpretation. *Nat Methods.* 2015;12:966-8.

2. DePristo MA, Banks E, Poplin R, et al. A framework for variation discovery and genotyping using next-generation DNA sequencing data. *Nat Genet.* 2011;43:491-8.

3. Wang K, Li M, Hakonarson H. ANNOVAR: functional annotation of genetic variants from high-throughput sequencing data. *Nucleic Acids Res.* 2010;38:e164.

4. Jumper J, Evans R, Pritzel A, et al. Highly accurate protein structure prediction with AlphaFold. *Nature.* 2021;596:583-589.
